# Analysis of transcript and protein overlap in a human osteosarcoma cell line

**DOI:** 10.1186/1471-2164-11-684

**Published:** 2010-12-02

**Authors:** Daniel Klevebring, Linn Fagerberg, Emma Lundberg, Olof Emanuelsson, Mathias Uhlén, Joakim Lundeberg

**Affiliations:** 1 Science for Life Laboratory, School of Biotechnology, Division of Gene Technology, Royal Institute of Technology, SE-171 65 Solna, Sweden; 2 Science for Life Laboratory, School of Biotechnology, Division of Proteomics, Royal Institute of Technology, SE-171 65 Solna, Sweden; 3 Department of Medical Epidemiology and Biostatistics, Karolinska Institute, Stockholm, Sweden

## Abstract

**Background:**

An interesting field of research in genomics and proteomics is to compare the overlap between the transcriptome and the proteome. Recently, the tools to analyse gene and protein expression on a whole-genome scale have been improved, including the availability of the new generation sequencing instruments and high-throughput antibody-based methods to analyze the presence and localization of proteins. In this study, we used massive transcriptome sequencing (RNA-seq) to investigate the transcriptome of a human osteosarcoma cell line and compared the expression levels with *in situ *protein data obtained in-situ from antibody-based immunohistochemistry (IHC) and immunofluorescence microscopy (IF).

**Results:**

A large-scale analysis based on 2749 genes was performed, corresponding to approximately 13% of the protein coding genes in the human genome. We found the presence of both RNA and proteins to a large fraction of the analyzed genes with 60% of the analyzed human genes detected by all three methods. Only 34 genes (1.2%) were not detected on the transcriptional or protein level with any method. Our data suggest that the majority of the human genes are expressed at detectable transcript or protein levels in this cell line. Since the reliability of antibodies depends on possible cross-reactivity, we compared the RNA and protein data using antibodies with different reliability scores based on various criteria, including Western blot analysis. Gene products detected in all three platforms generally have good antibody validation scores, while those detected only by antibodies, but not by RNA sequencing, generally consist of more low-scoring antibodies.

**Conclusion:**

This suggests that some antibodies are staining the cells in an unspecific manner, and that assessment of transcript presence by RNA-seq can provide guidance for validation of the corresponding antibodies.

## Background

Several studies have attempted to compare protein and transcript expression levels to investigate the central dogma of the cell, i.e. the relation between DNA, RNA and protein content in a cell [1-8]. Microarrays have been the prevalent platform to measure the abundance of transcripts in a sample, although other technologies such as SAGE have also been employed. The corresponding protein abundance estimates have frequently been obtained through mass spectrometry or protein arrays. The resulting correlation coefficients in these comparative analyses have varied significantly, from 0.3 to 0.9, comparing 10 s of genes up to 1000 s of genes. Sub-groups representing functionally different Gene Ontology groups could, however, display both higher and lower correlations depending on their role in the cellular machinery [7].

To improve an estimate of correlation between RNA and protein molecules a more unbiased approach combined with a digital gene expression profile is needed. Massive DNA sequencing technology offers a new possibility to achieve a comprehensive and quantitative view of all genes being transcribed in a sample [9-11]. Here, we compare global IHC and IF protein expression in a human osteosarcoma cell line, U-2 OS (from the Human Protein Atlas program, HPA)[12], with massive DNA sequencing of the corresponding transcriptome (RNA-seq).

## Results

The aim of this study was to compare the transcriptome of human U-2 OS cells with presence of the corresponding proteome. The transcriptome was extensively surveyed close to saturation by performing massive SOLiD DNA sequencing [Additional file 1: Supplemental figure S1]. In total, approximately 15 million high quality 35-bp reads were obtained and mapped onto the human reference genome (hg18) and quantitative measures were computed on a per gene basis. Analysis of the transcription pattern demonstrated that the majority of all Ensembl genes (73.4%; 15536/21146 genes) were expressed in U-2 OS, i.e., a transcript being represented by at least one uniquely mapped read. The frequency distribution is presented in the additional information [Additional file 1: Supplemental figure S2].

**Figure 1:**
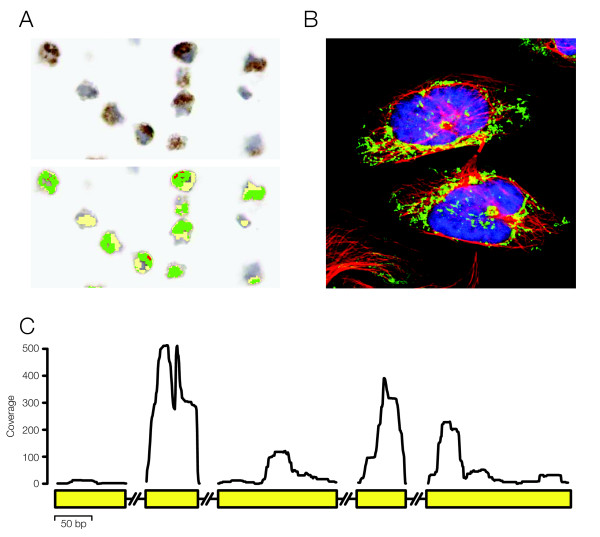
**Overview of the data types used**. (A) Images acquired from IHC were automatically processed and annotated. (B) Images from IF were manually annotated with staining intensity and a validation score. (C) For RNA-sequencing, reads mapping uniquely to exons were counted and an RPKM value was calculated for each gene.

To create a comparative protein expression set, a non-redundant collection of antibodies and genes was assembled from the Human Protein Atlas [12]. In the initial collection of data, a high degree of protein presence was observed for both IHC and IF, demonstrating expressed proteins for 88.7% and 73.6% of all genes analyzed, respectively (Table 1). In the following analysis, all antibodies with protein expression data from both IHC and IF in the U-2 OS cell line were used. For the genes with more than one antibody directed towards the gene product, the best scoring IF antibody was selected according to a standard validation scheme [Additional file 1: Supplemental table S1]. The assembled non-redundant set of antibodies was then used to collect corresponding immunohistochemistry and immunofluorescence information from U-2 OS, yielding the HPA subset. The HPA subset consists of 2749 Ensembl genes (with corresponding 2749 antibodies) that all have protein presence/absence information from both IHC and IF experiments (in the U2-OS cell line). Figure 1 shows the obtained data for gene *NDUFS4 *as an example of the input data for the three included platforms, IHC (A), IF (B) and RNA-seq (C).

**Table 1:** Study summary.

Method	Number of antibodies	Number of genes analyzed	Percentage of total number of genes analyzed	Number of genes present of common subset	Percentage present of common subset
**RNA-seq**	na	na	na	2345	85.3%
**IHC**	5329	4380	21.2%	2439	88.7%
**IF**	3626	3268	15.9%	2023	73.6%
**Common subset**	2749	2749	13.3%	na	na

### Protein distribution and overlap with transcriptional data

**Figure 2:**
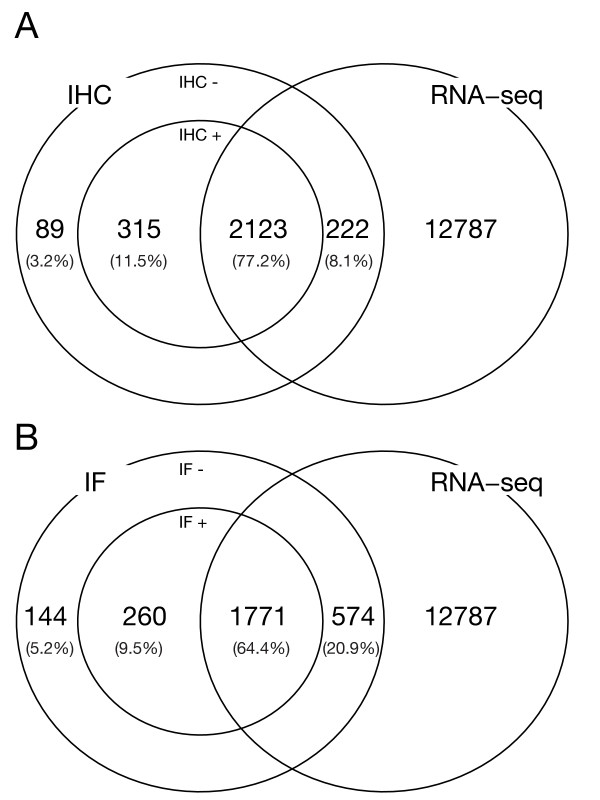
**(A) Overlap of IHC data with RNA data**. In IHC, 88.7% of the investigated genes are present. (B) As in (A), but overlap between IF and RNA-sequencing. In IF, 73.9% of the investigated genes are called present. The 'present overlaps' between IHC and RNA-sequencing and IF and RNA-seq are 77.2% and 64.4%, respectively.

Using the transcriptome sequencing strategy, we detect transcripts for 85.3% (2123+222) of the genes in the HPA subset (Table 1). A large overlap in expression is obvious comparing RNA-seq and the immunological assays. Figure 2 compares presence of proteins and transcripts, and it demonstrates that out of the HPA subset, RNA-seq detects 87.1% (2123/2123+315) of the IHC-detected proteins, and 87.2% (1771/1771+260) of the IF-detected proteins. These numbers are higher than what is expected by chance; a chi-square test results in p-values of 3.4 × 10^-13 ^and 2.6 × 10^-6 ^for IHC and IF, respectively. This supports a strong association between RNA and protein expression. The fact that approximately 13% of all detected proteins does not have a detectable transcript can indicate several different phenomena: (i) Some genes are very lowly expressed as transcripts, but efficiently translated into stable protein products or (ii) Some antibodies are cross-reactive, yielding false positive protein detection.

Interestingly, 9.4% (222/2123+222) of the genes in the HPA subset that were detected on the transcript level are not detected on the protein level. For IF, this number is 24.4% (574/1771+574). It is not clear if this is caused by a subset of genes that are transcribed but not translated, or if this is due to a limited sensitivity in the protein measurements. The fact that this fraction is higher for IF than IHC indicates that unspecific antibody-protein interaction in IHC in combination with a group of transcripts that do not undergo translation is the major contributor to this affect, since the sensitivity of IF is generally higher than that of IHC (see below).

### Comparison over three technology platforms

**Figure 3:**
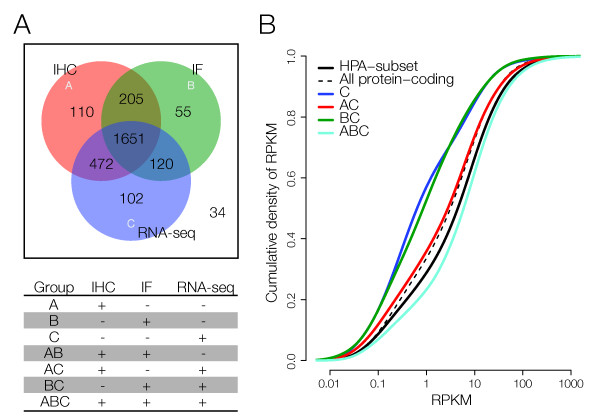
**(A) Venn diagram of presence flags for the three platforms (A = IHC, B = IF, C = RNA-seq)**. 60.1% of all genes investigated are present in all platforms. Only 34 genes (1.2%) are absent in all platforms. (B) Cumulative transcription density curves for the categories where RNA-sequencing data are available (C, AB, BC, ABC). A left-shifted curve contains a larger fraction of low transcribed genes. The category C (blue line) (RNA-seq only) contains genes with lower transcription than the full HPA subset (solid black line) (KS-test, one-sided, p = 3.8 × 10^-7^). Also, genes in BC (green line) (RNA-seq positive, IF positive, IHC negative) have generally a lower level of transcription than the HPA subset (KS-test, one-sided, p = 4 × 10^-5^). This is likely due to a higher sensitivity in IF than IHC. Interestingly, genes in the HPA subset display generally higher transcription levels (KS-test, one-sided, p < 2.2 × 10^-16^) than all protein coding genes (dashed black line).

For a more in-depth analysis, we investigated the expressed genes in a combined analysis of IHC, IF and RNA sequencing of the HPA subset (2749 genes). We show that 60.1% (1651 genes) of all investigated genes are detected by all platforms (Figure 3A) and only 1.2% (34 genes) was not detected by any platform. If only one of the two proteins detection platforms is required to call presence on the protein level (in the case of one of them producing a false positive call), only 3.2% - 5.2% of all genes are not detected on the transcript or RNA level. Interestingly, 71% (1651+205)/(110+472+1651+205+55+120) of proteins detected by either IHC or IF were detected by both methods. In total, IHC detects more proteins than IF (2438 vs. 2031). The higher number of detected genes likely indicates a higher degree of false positives, since genes detected by IF and RNA-seq are more lowly expressed than genes detected by IHC and RNA-seq (Figure 3B, see below).

Since RNA-seq provides quantitative measures of gene expression levels, we investigated transcript levels for the genes in the subgroups defined in Figure 3A, where this was possible (Figure 3B). This showed that the groups 'C' and 'BC' (detected in RNA-seq only and detected in IF and RNA-seq positive, respectively) were expressed at significantly lower levels than all genes combined (Kolmogorov-Smirnov (KS) test, p = 3.8 × 10^-4 ^and p = 4 × 10^-4^, respectively). This indicates that IF has a higher sensitivity to detect transcriptionally low expressed genes than IHC.

Next, we investigated the overlap between RNA and protein expression using the quantitative RPKM [10] values as a measure of transcript abundance. This measure is calculated by counting all reads that map to the exons of a gene and dividing by the length of the gene and total number of reads and is an expression value for each gene. The HPA subset was binned in 25 transcriptional levels, ranging from the top 5% of the bottom 5% expressed genes, as well as two transcriptional levels: the upper 50% and lower 50% expressed genes. Table 2 shows a 95% overlap between the upper 50% genes and protein presence based on IHC expression. For the lower 50%, this number drops to 82% and for IF, this overlap is 80% and 68% for the upper and lower intervals, respectively. Interestingly, for the smaller bins, this effect is very similar: The overlap is high (98% for IHC, 86% for IF) for the top 5% and remains relatively similar across the top 50% [Additional file 1: Supplemental figure S3]. Furthermore, we chose the subset of antibodies that had the highest validation score (supportive staining) in Western blot [Additional file 1: Supplemental table S1], and as expected, this yielded a slightly higher degree of overlap for both IHC and IF (Table 2) suggesting that some of the antibodies with a low validation score might be false positives.

**Table 2:** Overlap between RNA and protein based on RNA expression bins.

	Percent of present genes			
	
	All antibodies		Antibodies with supportive Western blot	
Fraction of genes based on transcript level	IHC	IF	IHC	IF
**Top 50%**	95.2%	79.9%	96.6%	83.1%
**Bottom 50%**	82.1%	67.8%	83.4%	73.2%

### Gene Set Enrichment Analysis

DAVID [13,14] is a tool that performs gene set enrichment analysis for several different categories (GO, KEGG, protein domains etc) and has the option to group similar categories into functional groups based on similarity. When the HPA subset (2749 genes) was analyzed for enrichment of gene categories against a background of all protein coding genes using DAVID some Gene Ontology categories emerged as over-represented. These include development, apoptosis, proteins related to direct protein sequencing, the cytoplasm and protein binding (data not shown).

Thus, since the HPA subset is somewhat biased from a gene category perspective, further gene set analysis of the subgroups was done with the HPA subset as background. We noticed that for genes in the ABC group (detected by all platforms, Figure 3A), certain themes were enriched. Two category sub-clusters were significantly over-represented; intracellular proteins and nuclear proteins (Table 3). From a technical perspective, these proteins are located within the cells (or even within the nucleus) and are therefore equally well detected using either IHC or IF. As a contrast, in the BC group (IHC-, IF+, RNA+) we find that extracellular proteins are significantly enriched (a group of proteins usually not detected by IF). Given the higher sensitivity of IF, we might speculate that these are proteins destined for export that still reside within the cells, and thus are present at very low levels.

**Table 3:** DAVID enrichment for certain categories

Group	Enriched theme	p-value
ABC	Nucleus	1, 03 × 10^-14^
	Intracellular	8,22 × 10^-23^
AB	Glycosylation site:N-linked (GlcNAc...)	9,97 × 10^-6^
BC	Extracellular region	4,39 × 10^-7^

**Figure 4:**
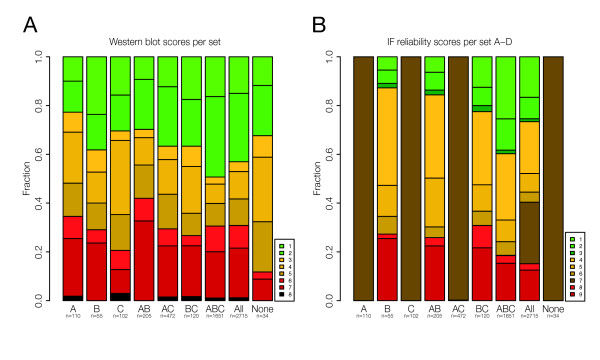
**(A) Western blot data for the groups defined in figure 3A**. The groups A and AB generally contain a larger fraction of low-scoring antibodies. (B) IF Reliability scores for the same subgroups. The fraction of antibodies with a supportive staining in the ABC group is about three times higher that in the B group (p < 2 × 10^-3^).

In the AB group (IHC+, IF+, RNA-), we notice that proteins related to glycosylation are significantly enriched. The apparent lack of correlation between RNA and protein expression for this GO category is not fully understood and requires further analysis to elucidate (Figure 4A, see below).

### Western blot and IF validation score analysis

The Western blots performed within the HPA program are manually investigated and assigned a validation score based on the number of detected bands, approximate size of the bands etc [Additional file 1: Supplemental table S1] using a standardized protein lysate panel. We analyzed these scores for all groups defined in Figure 3A. We observe that antibodies raised against genes in the A group (positive only in IHC) or the AB group (IHC+, IF+, RNA-) generally contain more low-scoring antibodies (KS-test, p = 1.6 × 10^-4 ^and KS-bootstrap test, p = 2 × 10^-3^) (Figure 4A). This suggests that some of these antibodies are staining the cells in an unspecific manner (false positives) and the RNA data can thus provide guidance for the validation of the corresponding antibodies. Western blot data was not available for our particular U-2 OS cell line analysed in this study, so a direct expression comparison between WB and other methods was not possible.

For IF images in the HPA program, a validation score is added after manual investigation. Comparative analysis of these scores could only be done for groups with staining according to IF, since a validation score of 7 is used to define absence. In the ABC group (present in all platforms), the fraction of antibodies receiving a supportive score [Additional file 1: Supplemental table S1] is about three times higher than that in the B group (present only in IF) and is confirmed significant (KS-bootstrap test, 2 × 10^-3^).

## Discussion

A quantitative comparison of the transcriptome and the proteome in a cell (or tissue) is instrumental in deciphering fundamental regulatory pathways and mechanisms. The relationship between these two sets of biomolecules also has wide implications for the identification of new biomarkers and classifiers in the treatment of disease. Previous efforts to compare the transcript and protein abundance, at single or multiple gene levels, have demonstrated a great variation in outcome, attributed to both biological and technical issues [1-8]. From a technical perspective, transcriptome monitoring has recently been greatly improved by RNA-seq, which provides a digital and comparably unbiased profile of all genes transcribed in a cell. We show that almost 75% of all genes are represented by one or more transcripts, which is in line with other recent studies [10]. We also demonstrate that lowly expressed genes are less likely to be identified at the protein level.

The proteome has in most cases been represented by indirect methods such as arrays or by mass spectometry of a solubilized proteome. Here, we present a large-scale comparison of in-situ protein abundance in cells with the transcription levels provided by RNA-seq. The proteins are assessed by antibodies targeting the proteome, and the presence of a particular protein species is visualized by immunohistochemistry and immunofluorescence microscopy, from which an abundance is estimated. These are two semi-quantitative methods and we therefore performed the comparison at the level of presense/absense.

For 87% of all detected proteins, we were able to detect a corresponding transcript. For the remaining 13% of all detected proteins (where we found no transcript), it can indicate several different phenomena: 1) Some genes are very lowly expressed as transcripts, but efficiently translated into stable protein products or 2) Some antibodies are cross-reactive, yielding false positive protein detection. Both of there effected would yield result where proteins are detected but the transcript is not.

We observe approximately 2/3 overlap between the RNA and its corresponding protein species, irrespective of method. Even if one of the two methods for generation of protein expression data is considered false, only 3.2% - 5.2% of all investigated genes are not detected on the protein or transcript level. Overall, immunohistochemistry identifies slightly more proteins than immunofluorescence, and we suggest that this elevated detection is in part due to false IHC positives, as indicated by the fact that IF demonstrates higher sensitivity for the lowly expressed genes (Figure 3B). Interestingly, the transcriptome-negative but IF- and IHC-positive group (8%), contained an over-representaion of genes related to glycosylation and needs to be confirmed with alternative methods such as mass spectrometry and RT-PCR. The group of genes detected by RNA-seq and IF but not IHC is significantly enriched for secreted proteins. Of 69 genes in this GO category, 33 are present in this group (p = 4.4 × 10^-7^).

## Conclustions

Our study demonstrates that RNA-seq is useful as a validation tool for the HPA program. In the case of previously uncharacterized proteins or conflicting data between protein array, Western blot, IHC and IF, we could use both the presence/absence call and the quantitative estimation of RNA molecules to decide on the quality of data. Indeed, presence of RNA does indicate that a corresponding protein can be expected in the analyzed sample. Furthermore, RNA-seq has been shown to efficiently identify splice variants [15], and this may also be used to discriminate between multiple antibodies directed towards different parts of the target protein.

We used the quantitative estimation of RNA molecules to show that the highest expressed genes are more likely represented by a corresponding protein. For the 50% highest expressed genes we detect the corresponding protein in more than 80% (IF) or 95% (IHC) of the cases. These numbers reveal a signficantly closer relationship between the presence of RNA and protein than what has been seen in previous studies. We believe that our data indicate that both RNA and proteins are expressed to a more significant extent than previously anticipated and that this means that cells are regulated at the level of protein abundance rather than on mere presence/absence. The next step in our understanding of the cell machinery will therefore require more sensitive and quantitative measures of proteins.

## Methods

### Cell cultivation

The osteosarcoma cell line U-2 OS (ATCC-LGC Promochem, Borås, Sweden) was cultivated in a 5% CO_2 _environment at 37°C in McCoy's 5A media, as suggested by the provider, with the addition of 10% Fetal Bovine Serum (FBS) and an antibiotic/antimycotic solution (both from Invitrogen).

### RNA sample preparation and cDNA synthesis

Cells were harvested and RNA was extracted using the RNeasy extraction kit as instructed by the manufacturer (Qiagen) and quality-assessed using the RNA nano kit on a BioAnalyzer 2100 (Agilent). The BioAnalyzer can interpret the generated data and score it with a RNA Integrity Number (RIN) ranging from 1 (very degraded) to 10 (no degradation). 10 μg of high-quality (RIN >9.5) total RNA was used as input material for depletion of ribosomal fragments using RiboMinus (Invitrogen). 250 ng ribosome-depleted RNA was adjusted to 4.5 μl in nuclease-free water and fragmented in 95°C for 20 minutes, after which it was immediately transferred to ice. 1 μl of biotinylated tagged random hexamers (Biotin-TEG-CTTTCCTCTCTATGGGCAGTCGGTGATNNNNNN, 1 pmol/μl, Operon) was added and the mixture was denatured at 70°C for 10 minutes and on ice for 2 minutes. During incubation, a cDNA-synthesis master mix consisting of (per sample) 6 μl 5x First-strand buffer (Invitrogen), 3 μl 0.1 M DTT (Invitrogen), 7.5 μl dNTP mixture (2 mM/dNTP) and 6.5 μl nuclease-free water was assembled on ice. Of this master mix, 23 μl was added to the denatured RNA:hexamer mixture together with 2 μl SuperScript III (Invitrogen) on ice. The first strand cDNA synthesis reaction was incubated at 20°C for 10 minutes followed by 37°C for 10 minutes and 42°C for 45 minutes. First-strand cDNA was purified by addition of 70 μl nuclease-free water using a MinElute spin column following the manufacture's instructions (Qiagen). Elution was carried out twice, each in 10 μl of EB-buffer, with a 180° rotation of the column in the centrifuge between the elutions. A second strand synthesis master mix was assembled on ice. This consisted of (per sample) 79 μl nuclease-free water, 30 μl 5x second-strand buffer (Invitrogen), 15 μl dNTP mixture (2 mM/dNTP), 1 μl 10 U/μl E. coli DNA Ligase (Invitrogen), 4 μl 10 U/μl E. coli DNA polymerase I (Invitrogen) and 1 μl 2 U/μl RNase H (Invitrogen). Of this, 130 μl was added to the ≈18 μl eluate containing the RNA:cDNA hybrid. The reaction was incubated at 16°C for 2 hours, after which 1.5 μl 3 U/μl T4 DNA Polymerase (New England Biolabs) was added, and the reaction was incubated for 5 more minutes at 16°C.

### Enrichment and SOLiD DNA sequencing

20 μl of Streptavidin Dynabeads M-270 (Invitrogen) were washed in 50 μl 1× BW-buffer (20 mM Tris, 2 mM EDTA, 1 M NaCl) and pelletized using a MPC-6 magnetic particle concentrator (Invitrogen). To the pellet, the 150-μl second-strand syntheis reaction and 150 2× BW buffer were added, mixed by gentle vortexing and incubated on gentle rotation using a RotaMix (Elmi) at room temperature (≈22°C) for 15 minutes. The bead-DNA complex was then washed three times in 100 μl sterile deionised water and pelletized. A PNK-master mix consisting of (per reaction) 15 μl sterile deionised water, 2 μl PNK buffer (Invitrogen), 2 μl 10 mM ATP and 1 μl 10 U/μl PNK was assembled on ice and 20 μl was added to the pelletized beads and mixed by pipetting. The reaction was incubated at room temperature for 15 minutes. To create a blunt-ended dsDNA adapter sequence suitable for ligation to the beads, a mixture consisting of 440 μl sterile deionised water, 5 μl 100 pmol/μl RDV primer (AACTGCCCCGGGTTCCTCATTCTCT, MWG-Biotech), 5 μl 100 pmol/μl aRDV primer (AGAGAATGAGGAACCCGGGGCAGTT, MWG-Biotech) and 50 μl PNK buffer (Invitrogen) was assembled and incubated at 95°C for 3 minutes and allowed to cool to room temperature on a lab bench for 30 minutes. A ligation master mix consisting of 4 μl 5× Ligase buffer (Invitrogen), 1 μl 1 pmol/μl RDV:aRDV duplex, 14 μl sterile deionised water and 1 μl 3 U/μl T4 DNA Ligase was assembled on ice, added to the pelletized beads and mixed by pipetting. The reaction was incubated for 16 hours on a RotaMix at room temperature. The ligation reaction was washed three times in sterile deionised water and the beads were resuspended in 20 μl of sterile deionised water.

An amplification master mix was assembled, consisting of 5 μl 5× HF buffer (Finnzymes), 10 μl sterile deionised water, 5 μl dNTP mix (2 mM/dNTP), 1 μl 10 pmol/μl RDV primer (AACTGCCCCGGGTTCCTCATTCTCT, MWG-Biotech), 1 μl 10 pmol/μl LAmpFDV (CCACTACGCCTCCGCTTTCCTCTCTATGGGCAGTCGGTGAT, MWG-Biotech) and 1 μl 2 U/μl Phusion polymerase (Finnzymes). 23 μl amplification master mix and 2 μl beads were mixed denatured at 95°C for 30 seconds and cycled as follows: 30 seconds at 95°C, 30 seconds at 55°C, 30 seconds at 72°C for 16 cycles. After a final extension at 72°C for 10 minutes, the PCR product was purified using a MinElute column, following the manufacturers instructions with the elution step as described earlier.

The PCR product was subjected to emPCR and SOLiD sequencing following the manufacturers instructions (Life Technologies/Applied Biosystems). Fifteen million 35-base pair reads passed quality filters including filtering against adaptors.

### Mapping of DNA reads and defining the transcriptome

The reads were mapped to the human genome (hg18) following the manufacturers instructions. A read was considered to be unique if is mapped to a single location with N mismatches and nowhere with N+1 or N+2 mismatches (i.e. a clear zone of 2). In total, 6 442 847 reads aligned uniquely to EnsEMBL genes. Only unique reads with a maximum of three mismatches were used to calculate expression values. A gene was considered present if at least one read fell entirely inside an exon of the gene. Raw sequence data has been deposited to the NCBI Short Read Archive with accession number SRA023713.1.

### Production of Antibodies

The antibodies used in this study have been generated within the Human Protein Atlas program http://www.proteinatlas.org[16]. For each application using the generated antibodies, a standard set of categories has been established, and these are grouped into three main validation scores: (i) supportive, (ii) uncertain, or (iii) non-supportive [Additional file 1: Supplemental table S1] [18].

### Western blot analysis

Prior to immunohistochemistry and immunofluorescence the HPA antibodies were analysed by Western blot as previously described [16]. All membranes were incubated with the primary antibodies diluted 1:500. The secondary HRP-conjugated antibody (Swine Anti-Rabbit Immunoglobulin/HRP, DakoCytomation) was diluted 1:3000 and detection was carried out using a CCD-camera. Seven categories (1-87) for the Western blot validation have been proposed in which three (grade 1-3) are *supportive*, two (grade 4-5) are *uncertain*, and two (grade 6-7) are *non-supportive*.

### Immunohistochemical analysis

Immunohistochemistry was performed on cell microarrays (CMA) where U-2 OS cells were represented, as previously described [18]. In brief, cells were harvested, fixed in formalin and dispersed into agarose. After histoprocessing and paraffin embedding of the cell pellets resulting in donor blocks, duplicate 0.6 mm punches were sampled and put into one recipient CMA. 4 micron sections were subsequently cut and immunohistochemically stained using an Autostainer Plus instrument (Dako, Glostrup, Denmark). An automated slide-scanner system, Scanscope T2 (Aperio Technology, Vista, CA, USA) was used to image the stained sections, resulting in digital images representing separated cell spots from the CMA.

The images were analyzed by an automated image analysis software, TMAx (Beecher instruments, Sun Praire, WI, USA), which identifies cells, measures the immunostaining and counts the fraction of stained cells, all according to processing logic previously described [19]. The measured staining intensity was further categorized into a IHC graded scale (negative, weak, moderate and strong). Here, the analyzed protein was considered "absent" when the staining intensity score was negative and "present" when the staining intensity score was weak, moderate or strong.

### Immunofluorescence microscopy

Immunofluorescent stainings were performed as previously described [20]. Briefly, cells were seeded in 96-well glass bottom plates, fixed with paraformaldehyde and permeabilized with Triton X-100 before immunofluorescently stained. The entire procedure was automated using a pipetting robot. Besides the HPA antibody staining, organelle markers for microtubules, endoplasmic reticulum and nuclei were included. Image acquisition was performed manually using a LSM 510 Meta confocal laser-scanning microscope equipped with a 63x oil-immersion objective (Carl Zeiss GmbH, Jena, Germany). For each sample, two representative four-channel images were acquired. The laser power and detector gain were adjusted for each sample to obtain as good signal to noise ratios as possible and to use the entire dynamic range of the detector.

The images were visually inspected and annotated in terms of staining intensity and subcellular localization. A graded scale (negative, weak, moderate and strong) was used to categorize the staining intensity based on the used laser power and detector gain. For each antibody a validation score was set based on how well the observed subcellular localization agreed with information in the UniProt database. The IF validation scores consist of a nine-graded scale that can be merged into three main categories: *supportive*, *uncertain *or *not supportive *[Additional file 1: Supplemental table S1]. Here, the analyzed protein was considered "absent" when the validation score was 7 (no staining, see [Additional file 1: Supplemental table S1]) and "present" otherwise.

## Authors' contributions

DK and LF analysed the data. DK performed low-level analysis of the RNA-seq data. LF performed low-level analysis of the IF and IHC data. DK and JL wrote the manuscript. EL and OE interpreted data. MU and JL designed the study. All authors read and approved the final manuscript.

## Supplementary Material

Additional file 1 **Supporting tables and figures**. Supporting tables and figures. Click here for file
