# Supplementary material for: Analysis of transcript and protein overlap in a human osteosarcoma cell line
Source: BMC Genomics. 2010 Dec 2;11:684. doi: 10.1186/1471-2164-11-684 (PMC3014981; doi:10.1186/1471-2164-11-684)

# Supplemental Information

## Supplemental figure 1. Sequencing to saturation


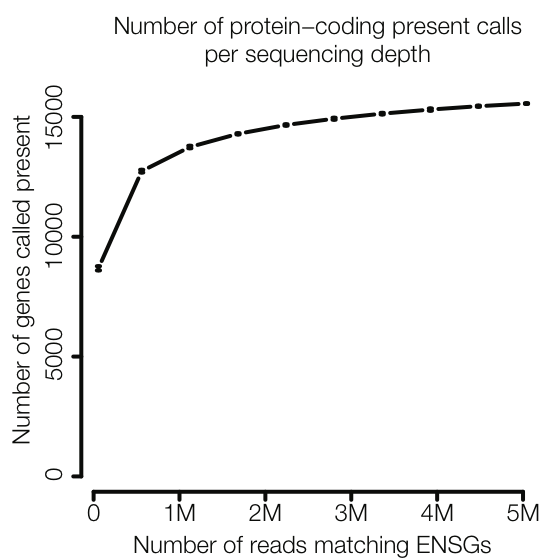


Subset of the complete data set were selected and used for approximation of the number of present genes. Using about 5 million reads, this number has reached a stable level.

## Supplemental figure 2. Histogram of gene expression values from RNA-seq


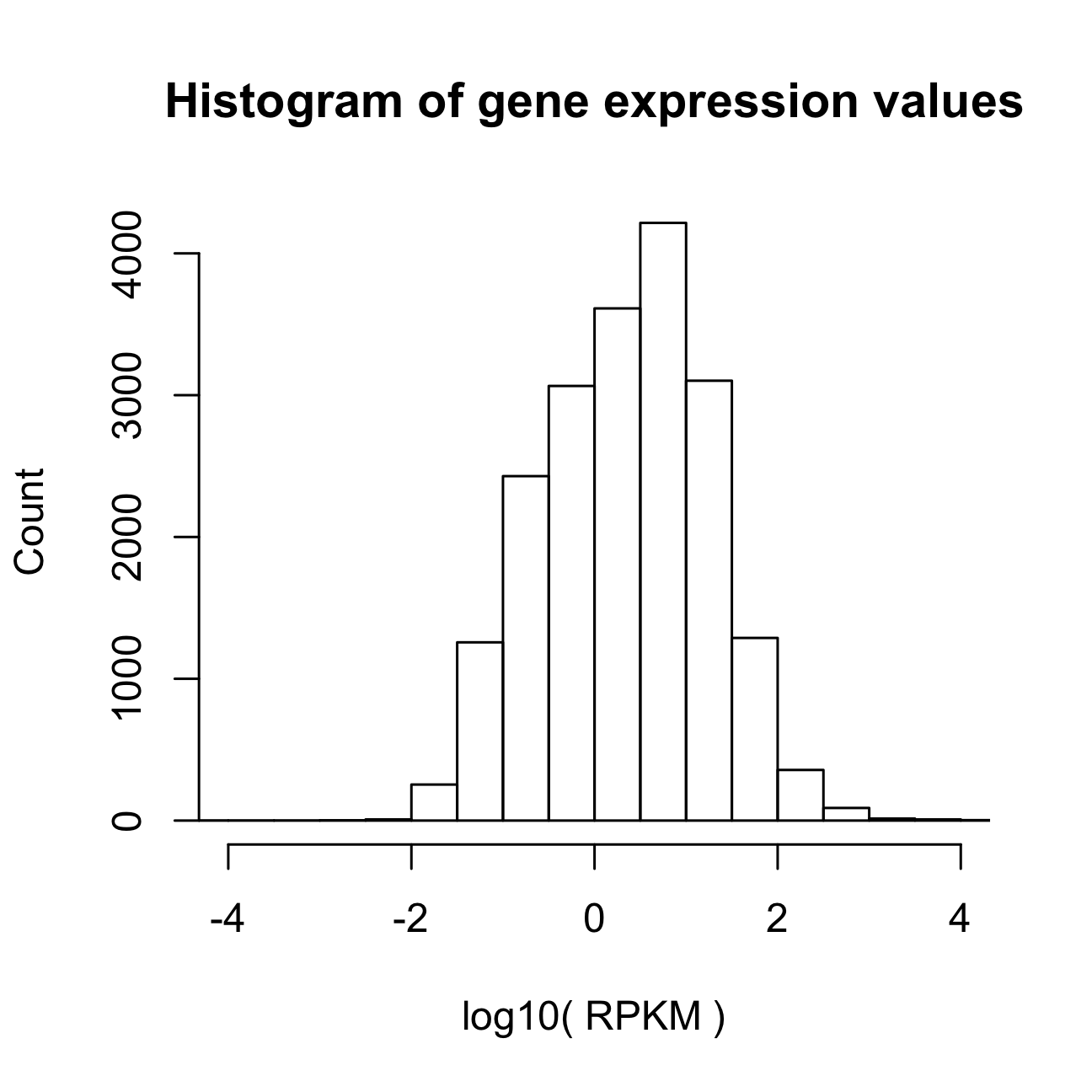


Distribution of RPKM gene expression values from RNA-seq.

## Supplemental figure 3. Percent overlap in different expression bins


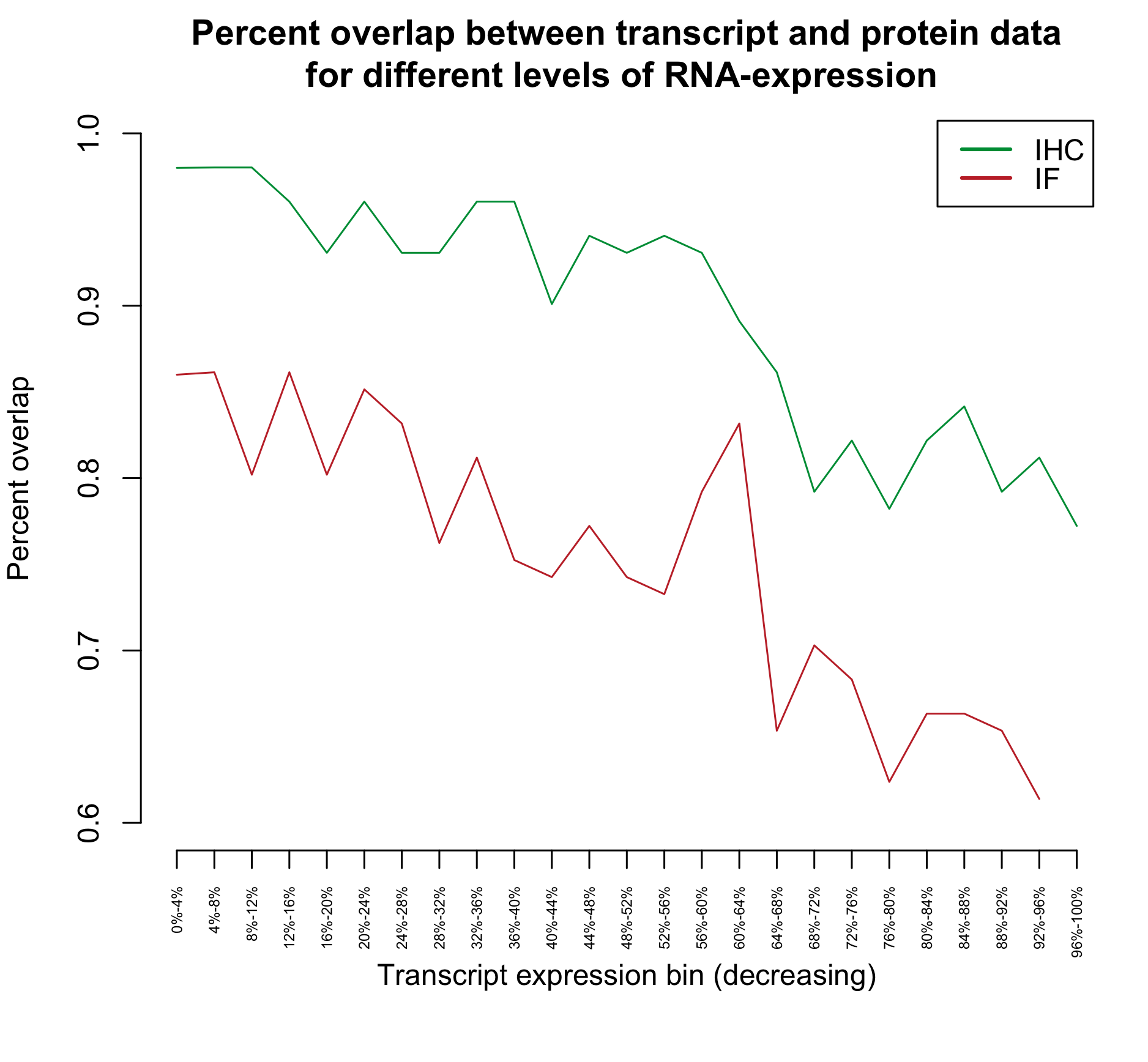


Percent overlap of present genes in expression bins. The leftmost bin corresponds to the top 5% expressed genes and the rightmost to the bottom 5%.

## Supplemental table 1


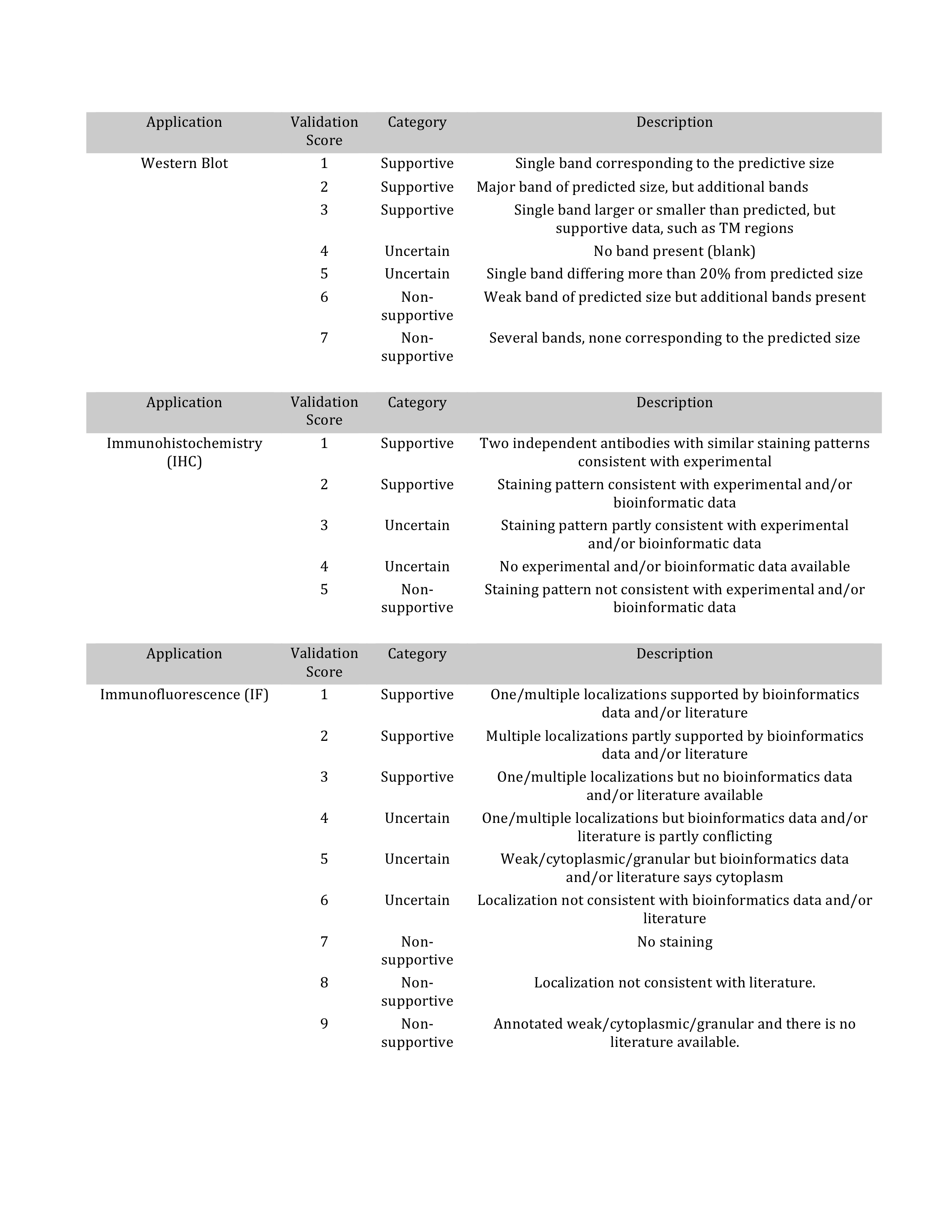

Supplement: Additional file 1 — Supporting tables and figures. Supporting tables and figures. [file 1471-2164-11-684-S1.DOC]
